# Thrombotic gene polymorphisms and postoperative outcome after coronary artery bypass graft surgery

**DOI:** 10.1186/1749-8090-6-120

**Published:** 2011-09-28

**Authors:** Ozan Emiroglu, Serkan Durdu, Yonca Egin, Ahmet R Akar, Yesim D Alakoc, Cagin Zaim, Umit Ozyurda, Nejat Akar

**Affiliations:** 1 Department of Cardiovascular Surgery, Nicosia State Hospital, Nalbantoglu Lefkosa Devlet Hastanesi, Ortakoy, Nicosia, Cyprus; 2 Department of Cardiovascular Surgery, Ankara University School of Medicine, Cebeci Kalp Merkezi, Dikimevi, Ankara, 06340 Turkey; 3 Department of Pediatric Molecular Genetics, Ankara University School of Medicine, Cebeci Tıp Fakültesi, Dikimevi, Ankara, 06340 Turkey

## Abstract

**Background:**

Emerging perioperative genomics may influence the direction of risk assessment and surgical strategies in cardiac surgery. The aim of this study was to investigate whether single nucleotide polymorphisms (SNP) affect the clinical presentation and predispose to increased risk for postoperative adverse events in patients undergoing coronary artery bypass grafting surgery (CABG).

**Methods:**

A total of 220 patients undergoing first-time CABG between January 2005 and May 2008 were screened for factor V gene G1691A (FVL), prothrombin/factor II G20210A (PT G20210A), angiotensin I-converting enzyme insertion/deletion (ACE-ins/del) polymorphisms by PCR and Real Time PCR. End points were defined as death, myocardial infarction, stroke, postoperative bleeding, respiratory and renal insufficiency and event-free survival. Patients were compared to assess for any independent association between genotypes for thrombosis and postoperative phenotypes.

**Results:**

Among 220 patients, the prevalence of the heterozygous FVL mutation was 10.9% (n = 24), and 3.6% (n = 8) were heterozygous carriers of the PT G20210A mutation. Genotype distribution of ACE-ins/del was 16.6%, 51.9%, and 31.5% in genotypes I/I, I/D, and D/D, respectively. FVL and PT G20210A mutations were associated with higher prevalence of totally occluded coronary arteries (p < 0.001). Furthermore the risk of left ventricular aneurysm formation was significantly higher in FVL heterozygote group compared to FVL G1691G (*p *= 0.002). ACE D/D genotype was associated with hypertension (*p *= 0.004), peripheral vascular disease (p = 0.006), and previous myocardial infarction (*p *= 0.007).

**Conclusions:**

FVL and PT G20210A genotypes had a higher prevalence of totally occluded vessels potentially as a result of atherothrombotic events. However, none of the genotypes investigated were independently associated with mortality.

## Background

Clinical significance of gene polymorphisms involved in haemostatic pathways including coagulation, fibrinolysis, platelet glycoprotein receptor function, and renin-angiotensin system on postoperative outcome following cardiac surgery is limited [1-4]. Currently available and widely used predictive models such as EuroSCORE, and the Society of Thoracic Surgeons National Adult Cardiac Surgery Database (STS NCD) for risk stratification in cardiac surgical practice often lack genomic risk factors. However, current thinking accords a primordial role to patients' individual responses to surgical stress, extracorporeal circulation, and pharmacologic interventions even in patients with similar comorbidities [5-8]. Furthermore, inherited single nucleotide polymorphisms (SNPs) related to the coagulation system have been reported as risk factors for venous thrombosis, ischemic stroke, coronary artery disease and myocardial infarction [9]. Much recent interest in cardiac surgery has centered on perioperative genomics that may improve risk assessment systems and outcome prediction in cardiac surgical population as well as appropriate perioperative management for patients with hypercoagulable states [6]. Therefore, it is possible that single genetic risk factors are involved in the occurrence of adverse events at different time points following cardiac surgery.

The aim of this observational study of all prospectively collected data was to investigate the possible links between factor V Leiden gene G1691A (FVL), prothrombin/factor II G20210A (PT G20210A), and angiotensin-converting enzyme gene insertion/deletion polymorphism (ACE-ins/del) and the preoperative clinical presentation in patients undergoing CABG. In addition, we aimed to assess whether these SNPs predispose to increased risk for post-operative adverse events. Polygenic nature of atherothrombosis was the rationale for the selection of more than one mutation or polymorphism.

## Methods

### Patients and Protocol

The Research Ethics Committee of the Ankara University School of Medicine approved the study protocol and all subjects gave written informed consent to participate in the study. The study was performed in accordance with the Helsinki declaration. We prospectively enrolled 220 consecutive adult patients (165 males and 55 females) with coronary artery disease (CAD) who were scheduled for coronary artery bypass grafting (CABG) using cardiopulmonary bypass (CPB). All subjects were born and living in Turkey. The decision for surgical revascularization was based on symptoms, presence of ischemia, echocardiography, single-photon emission computed tomography and coronary angiographic findings.

Patients were not enrolled in the study if any one of the following exclusion criteria were met: (1) life expectancy less than 2 years; (2) previous cardiac surgery; (3) off-pump CABG; (4) dialysis dependent renal failure; (5) hepatic dysfunction; (6) morbid obesity or cachexia; (7) use of oral contraceptives; (8) emergent or urgent surgery. Aspirin or clopidogrel therapies were discontinued at least 5 days before the scheduled date of the operation as part of routine care.

### Data collection and definitions

Baseline, procedural, and follow-up data were stored prospectively in a database located at the University of Ankara. Patients' preoperative risk factors were recorded and EuroScores were calculated for each patient. For all patients, we analyzed the following characteristics: age, gender, body surface area, positive family history of cardiovascular disorders, presence or absence of chronic or unstable angina, symptoms of heart failure, pulmonary hypertension, percutaneous coronary intervention, left ventricular ejection fraction (LVEF), smoking habits, presence of comorbidities, including diabetes, hypertension, hypercholesterolemia, peripheral vascular disease, history of stroke, renal function, elective versus urgent surgery. Intraoperative variables included number of coronary bypass grafts, duration of CPB, duration of aortic cross-clamp, requirement for inotropic drugs, and/or intra-aortic balloon pump counterpulsation, and blood product use. Postoperative data comprised myocardial infarction, tamponade, reoperation for occlusion or other causes, requirement of intra-aortic balloon pump support, neurologic complications, renal dysfunction, chest tube drainage during the first 24 postoperative hours, total chest tube drainage, the length of mechanical ventilator support, pneumonia, multiorgan failure, gastrointestinal complications, sepsis, coma, deep vein thrombosis, the length of intensive care unit (ICU) stay, and readmission within 90 days after surgery.

Adverse events were defined as death, perioperative myocardial infarction, stroke, re-exploration due to bleeding, respiratory insufficiency, and renal failure. Perioperative MI was defined as either new Q waves or ischemic ST segment changes with concomitant elevations of creatine kinase isoenzyme (CK-MB) > 5 times the upper limit of the reference range or a CK-MB to total creatine kinase ratio > 10% occurring within 48 hours after surgery or troponin I > 1 ng/mL. Renal dysfunction was defined as rise of serum creatinine above 2.5 mg/dL and/or a need for hemodialysis. The surgical team saw all patients about 4-6 weeks after discharge and annually thereafter for two years.

### Surgical and Postoperative Considerations

Preoperative work-up, anesthesia and cardiopulmonary bypass management were conducted according to our institutional protocol. All patients underwent cardiac surgery with non-pulsatile CPB by using roller pumps and disposable membrane oxygenators. The pump was primed with 1200 mL of lactated Ringer solution with 100 mmol of sodium bicarbonate and 5000 IU of heparin were added. CPB was instituted at a flow rate of 2.4 L/min/m^2 ^after systemic heparin administration (1 mg/kg). During CPB, the mean arterial pressure target was set at 60 mm Hg, and the core temperature of the patients was allowed to drift to 30-32°C during CPB. Alpha-stat pH management was employed. Intermittent cold-blood cardioplegia (1:4 blood to crystalloid with maximal potassium concentration 22 mEq/L) was delivered antegrade via the aortic root. At the conclusion of CPB, anticoagulation was reversed with protamine. Cross clamp, total perfusion times, and duration of the operation were recorded. None of the patients received epsilon amino caproic acid, tranexamic acid, and aprotinin. In postoperative course, patients received low molecular weight heparin (LMWH) and 300 mg of aspirin starting from second day of surgery. LMWH was continued until patients were mobilized. In suspicion of postoperative deep vein thrombosis colour doppler ultrasonography was performed to confirm the suspect diagnosis.

### Blood Collection and Laboratory Analysis for Genotyping

A preoperative morning fasting blood sample was drawn from the antecubital vein into 10 mL polypropylene tubes containing 1 ml Ethylene-Diamine-Tetra-Acetic acid (EDTA). Genomic DNA isolation was performed using standard phenol-chloroform extraction method. The FVL and the PT G20210A were determined according to a standardized real-time polymerase chain reaction (RT-PCR) method using Light Cycler Mutation Kits (Tibmolbiol Roche Diagnostics GmbH, Roche Molecular Biochemicals, Manheim, Germany) as previously described [10,11]. In order to determine the ACE-ins/del morphology PCR was used [12]. Amplification was performed for 35 cycles with an annealing temperature of 58°C (Biometra, USA). Amplified DNA was subjected on to 2.5% agarose gel electrophoresis and visualized by ethidium bromide staining. Ankara University Pediatric Molecular Genetics research laboratory personnel who were blinded from the clinical data performed genetic analyses.

### Statistical analyses

The primary end points were the effect of gene polymorphisms on postoperative mortality, perioperative myocardial infarction, and stroke. The secondary end points were re-exploration due to bleeding, respiratory insufficiency, renal failure and event-free survival. Differences in baseline, and clinical characteristics between carriers and unaffected patients were assessed by the chi-square test, t-test, or Fisher's Exact test, where appropriate. The Mann-Whitney test was used to calculate the mean for nonparametric variables. Statistical analyses were performed using a software program (SPSS 16.0 for Windows; SPSS Inc, Chicago, Ill). Predicted risk scores for in-hospital mortality were calculated using previously validated models of the EuroSCORE. The significance level for difference for all tests was *p *value less than 0.05.

## Results and Discussion

### Demographic Characteristics and Genotype Distribution

A total of 220 patients' (62.5 ± 10.2 years of age, range 43-74 years) blood samples were analyzed for FVL, G20210A genotype of prothrombin. We found no patients homozygous for FVL and PT G20210A. Among 220 patients, the prevalence of the heterozygous FVL mutation was 10.9% (n = 24), and 3.6% (n = 8) were heterozygous carriers for PT 20210G > A mutation. ACE-ins/del genotypes were studied in 181 patients. Genotype distribution of ACE-ins/del was 16.6%, 51.9%, and 31.5% in genotypes I/I, I/D, and D/D, respectively. The distribution of FVL, PT G20210A and ACE-ins/del genotypes are given in Table 1. The selected clinical characteristics of the study group are reported in Table 2. As expected the study population had a higher prevalence of traditional atherosclerotic risk factors at baseline. Follow-up information was complete in 218 patients (99.1%).

**Table 1:** Factor V Leiden, PT G20210A, and ACE-ins/del Genotype Frequencies in Coronary Artery Bypass Graft Surgery Patients.

Single nucleotide polymorphism (n)	(n)	(%)	Screened Patients (n)
FVL 1691			220
G/G	196	89.1	
G/A	24	10.9	
A/A	0	0	
PT 20210			220
G/G	212	96.4	
G/A	8	3.6	
A/A	0	0	
ACE-ins/del			181
I/I	30	16.6	
I/D	94	51.9	

FVL indicates factor V Leiden; PT 20210, prothrombin/factor II; ACE-ins/del, angiotensin-converting enzyme gene insertion/deletion polymorphism.

**Table 2:** Main Preoperative Characteristics of the Study Population.

	Study population (n = 220)	FVL 1691 G/G (n = 196)	FVL 1691 G/A (n = 24)	p value*	PT 20210 G/G (n = 212)	PT 20210 G/A (n = 8)	p value**	ACE I/I and ACE I/D (n = 124)	ACE D/D^+^ (n = 57)	p value***
Age (yrs), mean ± SD	62.5 ± 10.2	62.5 ± 10.2	62.3 ± 10.6	NS	62.7 ± 10.2	57.6 ± 8.5	NS	62.0 ± 9.7	63.2 ± 10.5	NS
Female sex, *n *(%)	58 (26.4)	54 (24.6)	4 (16.7)	NS	56 (26.4)	2 (25)	NS	29 (23.4)	13 (22.8)	NS
Body surface area (m^2^), mean ± SD	1.8 ± 0.2	1.8 ± 0.2	1.8 ± 0.1	NS	1.8 ± 0.2	1.8 ± 0.2	NS	1.8 ± 0.2	1.79 ± 0.14	NS
Hypertension, *n *(%)	89 (40.5)	80 (40.8)	9 (37.5)	NS	88 (41.5)	1 (12.5)	NS	46 (37.1)	34 (59.7)	0.004
Diabetes, *n *(%)	118 (53.6)	107 (54.6)	11 (45.8)	NS	113 (53.3)	5 (62.5)	NS	68 (54.8)	29 (50.9)	NS
Hypercholesterolemia, *n *(%)	94 (42.7)	83 (42.4)	11 (45.8)	NS	92 (43.4)	2 (25)	NS	59 (47.6)	28 (49.1)	NS
Smoking, *n *(%)	122 (55.5)	107 (54.6)	15 (62.5)	NS	122 (57.6)	2 (25)	NS	66 (53.2)	35 (61.4)	NS
Previous stroke, *n *(%)	3 (1.4)	3 (1.5)	0	NS	3 (1.4)	0	NS	1 (0.8)	1 (1.8)	NS
Peripheral arterial disease, *n *(%)	25 (11.4)	22 (11.2)	3 (12.5)	NS	24 (11.3)	1 (12.5)	NS	10 (8.1)	13 (22.8)	0.006
Previous hospital admission for MI, *n *(%)	52 (23.6)	43 (21.9)	9 (37.5)	NS	51 (24.1)	1 (12.5)	NS	26 (21.0)	23 (40.4)	0.007
COPD, *n *(%)	32 (14.6)	28 (12.7)	4 (16.7)	NS	32 (15.1)	0	NS	17 (13.7)	12 (21.1)	NS
Creatinine > 2.3 mg/dL, *n *(%)	7 (3.2)	5 (2.6)	2 (8.3)	NS	6 (2.8)	1 (12.5)	NS	1 (0.8)	2 (3.5)	NS
Catheter lab emergency, *n *(%)	5 (2.6)	5 (2.6)	0	NS	4 (1.9)	1 (12.5)	NS	0	2 (3.5)	NS
Number of diseased coronary arteries, *n *(%)	2.4 ± 0.7	2.4 ± 0.7	2.4 ± 0.7	NS	2.4 ± 0.7	2.4 ± 0.9	NS	2.4 ± 0.71	2.5 ± 0.7	NS
Totally Occluded Coronary Arteries, *n *(%)	0.1 ± 0.3	0.1 ± 0.2	0.46 ± 0.7	< 0.001	0.08 ± 0.3	0.3 ± 0.5	< 0.001	0.1 ± 0.35	0.1 ± 0.3	NS
Left ventricular aneurysm, *n *(%)	10 (4.6)	5 (2.6)	5 (20.8)	0.002	9 (4.3)	1 (12.5)	NS	8 (6.5)	1 (1.8)	NS
Intracardiac thrombus, *n *(%)	2 (0.9)	2 (0.9)	0	NS	2 (0.9)	0	NS	1 (0.8)	1 (1.8)	NS
Ischemic mitral regurgitation, *n *(%)	19 (8.6)	16 (8.2)	3 (12.5)	NS	17 (8.0)	2 (25)	NS	15 (12.1)	4 (7.0)	NS
LVEF (%) mean ± SD	52.8 ± 9.9	53.3 ± 9.9	49.3 ± 9.0	NS	52.7 ± 10.0	55.9 ± 4.9	NS	53.6 ± 10.1	52.84 ± 10.1	NS
EuroScore, mean ± SD	1.3 ± 1.3	1.9 ± 1.5	1.9 ± 1.5	NS	2.0 ± 1.5	1.9 ± 1.5	NS	1.85 ± 1.5	1.93 ± 1.4	NS

Values are mean ± SD or percentage of patients, FVL indicates factor V Leiden; PT 20210, prothrombin/factor II; ACE-ins/del, angiotensin-converting enzyme gene insertion/deletion polymorphism; COPD, chronic obstructive pulmonary disease; LVEF, left ventricular ejection fraction; NS, Non-significant. The study population of ACE D/D genotype consisted of 181 patients.

* FVL 1691G/G compared with FVL 1691 G/A polymorphism; ** PT 20210 G/G compared with PT 20210 G/A polymorphism; *** ACE I/I and ACE I/D group compared with ACE D/D polymorphism group.

FVL and PT G20210A polymorphisms were associated with higher incidence of totally occluded coronary arteries (*p *< 0.001). Furthermore the risk of left ventricular aneurysm formation was significantly higher in FVL group compared to FVL G1691G (*p *= 0.002). ACE D/D genotype had significantly increased association with hypertension (*p *= 0.004), peripheral vascular disease (*p *= 0.006), and previous MI compared to ACE I/I and I/D genotypes (*p *= 0.007).

### Postoperative Outcomes of Patients According Their Genotypes

None of the genotypes investigated were independently associated with excess mortality. Post-operative data is summarized in Table 3. The overall in-hospital mortality rate was 2.7% for all patients and was not significant between genotypes. There was no incidence of postoperative deep vein thrombosis in this cohort. Prevalence of primary and secondary study end-points for FVL, PT G20210A, and ACE-ins/del genotypes are shown in Table 4.

**Table 3:** Perioperative Variables and Postoperative Complications of the Study Population

	Study population (n = 220)	FVL 1691 G/G (n = 196)	FVL 1691 G/A (n = 24)	p value*	PT 20210 G/G (n = 212)	PT 20210 G/A (n = 8)	p value**	ACE I/I & I/D (n = 124)	ACE D/D (n = 57)	p value***
CPB time (min)	77.5 ± 15.7	77.5 ± 15.7	77.3 ± 15.9	NS	77.5 ± 15.9	77.5 ± 9.0	NS	79.2 ± 17.5	75.2 ± 11.5	NS
Number of coronary grafts	3.0 ± 1.1	3.0 ± 1.1	3.0 ± 0.9	NS	3.0 ± 1.1	2.4 ± 0.9	NS	3.1 ± 1.1	2.9 ± 1.0	NS
Postoperative blood loss at 24 hours, mL	463.5 ± 155.1	487.1 ± 150.5	460.6 ± 155.7	NS	459.6 ± 133.6	566.9 ± 449.0	NS	448.5 ± 136.0	470.9 ± 130.5	NS
Total drainage, mL	604.3 ± 204.1	597.4 ± 191.5	660.4 ± 286.3	NS	599.6 ± 176.5	730.0 ± 587.9	NS	581.1 ± 179.8	631.9 ± 187.4	0.045
Stay in intensive care unit, hours	54.9 ± 80.4	56.6 ± 85.0	42.0 ± 9.1	NS	52.9 ± 73.4	109.6 ± 190.3	NS	56.3 ± 82.7	53.0 ± 60.4	NS
In-hospital Mortality, %	6 (2.7)	6 (3.1)	0	NS	5 (2.4)	1 (12.5)	NS	3 (2.4)	2 (3.5)	NS

* FVL 1691G/G compared with FVL 1691 G/A polymorphism; ** PT 20210 G/G compared with PT 20210 G/A polymorphism; *** ACE I/I and ACE I/D group compared with ACE D/D polymorphism group.

**Table 4:** Prevalence of primary and secondary study end-points for FVL G1691A, PT G20210A, and ACE-ins/del Genotypes.

		FVL 1691 G/G (n = 196)	FVL 1691 G/A (n = 24)	*p *value	PT 20210 G/G (n = 212)	PT 20210 G/A (n = 8)	*p *value	ACE I/I (n = 30)	ACE I/D (n = 94)	ACE D/D (n = 57)	*p *value
Re-exploration due to bleeding, *n *(%)	No	193 (98.5)	23 (95.8)	NS	209 (98.6)	7 (87.5)	NS	29 (96.7)	93 (98.9)	55 (96.5)	NS
	Yes	3 (1.5)	1 (4.2)		3 (1.4)	1 (12.5)		1 (3.3)	1 (1.1)	2 (3.5)	

Perioperative acute MI, *n *(%)	No	194 (99.0)	24 (100)	NS	211 (99.5)	7 (87.5)	0.072	29 (96.7)	93 (98.9)	55 (96.5)	NS
	Yes	2 (1.0)	0		1 (0.5)	1 (12.5)		1 (3.3)	1 (1.1)	2 (3.5)	

Postoperative stroke, *n *(%)	No	194 (99.0)	24 (100)	NS	210 (99.0)	8 (100)	NS	30 (100)	93 (98.9)	57 (100)	NS
	Yes	2 (1.0)	0		2 (0.9)	0		0	1 (1.1)	0	

Respiratory insufficiency, *n *(%)	No	191 (97.5)	24 (100)	NS	207 (97.6)	8 (100)	NS	30 (100)	92 (97.9)	55 (96.5)	NS
	Yes	5 (2.6)	0		5 (2.4)	0		0	2 (2.1)	2 (3.5)	

Renal insufficiency, *n *(%)	No	191 (97.5)	24 (100)	NS	206 (97.2)	8 (100)	NS	30 (100)	92 (97.9)	55 (96.5)	NS
	Yes	5 (2.6)	0		5 (2.4)	0		0	2 (2.1)	2 (3.5)	

Event-free survival, *n *(%)	No	9 (4.6)	1 (4.2)	NS	9 (4.2)	1 (12.5)	NS	1 (3.3)	4 (4.3)	4 (7.0)	NS
	Yes	187 (95.4)	23 (95.8)		203 (95.8)	7 (87.5)		29 (96.7)	90 (95.7)	53 (93.0)	

Overall death at 5-years, *n *(%)	No	189 (96.4)	23 (95.8)	NS	205 (96.7)	7 (87.5)	NS	30 (100)	91 (96.8)	54 (94.7)	NS
	Yes	7 (3.6)	1 (4.2)		7 (3.3)	1 (12.5)		0	3 (3.2)	3 (5.3)	

FVL indicates factor V Leiden; PT 20210, prothrombin/factor II; ACE-ins/del, angiotensin-converting enzyme gene insertion/deletion polymorphism; MI, myocardial infarction; NS, non-significant

## Conclusions

Presence of classic risk factors for atherosclerosis, such as smoking, obesity, diabetes, hypertension, and hypercholesterolemia also affect the outcomes following percutaneous coronary interventions or CABG. In the present study, we investigated the genetic contribution of FVL, PT G20210A and ACE-ins/del gene polymorphisms to risk of adverse cardiovascular outcomes after CABG and preoperative clinical presentation. However, in this prospective study, we found no relation between any of the polymorphisms studied and the risk of mortality, perioperative myocardial infarction, and stroke or with postoperative renal and respiratory insufficiency. Although differences were not significant, a trend was noted for the PT G20210A genotype with postoperative MI (*p *= 0.072).

FVL, a common variant in the factor V gene resulting activated protein C resistance has a prevalence of 2 to 7 percent in most European populations and has been observed in 20 to 50 percent of patients with venous thromboembolic disease [13]. In this present study, we noted that heterozygous FVL polymorphism prevalence was 10.9% in patients undergoing surgical revascularization, which was compatible with 7.9% prevalence rate in healthy individuals (n = 4276) pooled from 26 centers in Turkey [14]. Strikingly higher prevalence rates of FVL among Turkish population in comparison to European series deserve attention for further meta-analysis of European populations. Currently, the risk of atherothrombotic events associated with the FVL polymorphism is controversial. In a meta-analysis of 191 studies involving a total of 66 155 cases and 91 307 controls, Ye *et al*. showed moderate but highly significant associations of coronary disease risk with the FVL and PT G20210A polymorphisms, both of which increase circulating thrombin generation [9]. Regarding surgical outcomes, however, Völzke *et al*. found no association between FVL and glycoprotein IIIa PlA1/PlA2 gene polymorphisms and mid-term mortality or cardiac morbidity after CABG [3]. Recently, Massoudy *et al*. have reported that the incidence of perioperative and postoperative thromboembolic events was high in cardiac surgical patients with symptomatic heterozygous FVL disease and suggested preoperative screening [15]. To assess the risk for poor surgical outcome associated with FVL, heterozygous carriers were compared with non-carriers. In contrast, the previous study, we did not observe a significant difference regarding fatal and nonfatal thromboembolic events in the postoperative period, which may be due to routine use of low molecular weight heparin postoperatively [15]. It should also be noted that one patient with FVL polymorphism from our study cohort developed fatal mesenteric ischemia postoperatively. Donahue *et al*. showed that heterozygous FVL polymorphism was associated with significantly lower blood loss and decreased risk for transfusion at 6 and 24 hours postoperatively in cardiac surgical patients [16]. We were, however, unable to confirm the previously reported protective effect of FVL polymorphism on post-surgical blood loss in our series.

Hereditary autosomal dominant prothrombin G20210A was discovered in 1996 [17]. We have previously reported that PT 20210 A allele frequency in Turkish population was 2.7% in healthy controls and 6.3% in patients with deep vein thrombosis. The present study confirms that PT 20210 A allele frequency in Turkish patients undergoing CABG surgery is 3.6%. Doggen *et al*. from Leiden University demonstrated that the prevalence rate of heterozygous carriers of the 20210 variant of the PT gene was 1.8% among 560 men with a first myocardial infarction before the age of 70 years [18]. Furthermore, they showed that thrombotic risk factors, such as PT G20210A and FVL polymorphism, increase the risk of myocardial infarction in men by 1.4. FVL and PT G20210A carriers in this study had higher prevalence of totally occluded coronary arteries and left ventricular aneurysm formation, which constitutes an original finding about the correlation of two aforementioned SNPs and coronary atherothrombosis. We suggest that formation of thrombosis in atherosclerotic coronary arteries leading to total occlusion and left ventricular aneurysm is more likely in FVL and PT G20210A carriers. The findings of a recent study regarding the increased frequencies of FVL or prothrombin variant G20210A in patients age < 50 years who suffer MI but have no significant coronary stenosis at angiography supports our atherothrombosis hypothesis in FVL and PT G20210A carriers [11]. Furthermore, a pooled analysis of two studies which provided genotype frequencies in patients with no, one, two, or three vessel disease showed, a greater prevalence of the PT G20210A genotype among patients with no or one vessel disease than in those with multi-vessel disease (4.4% vs 2.2%) suggesting that the 20210A prothrombin gene variant may be a significant genetic factor for hypercoagulability in patients with ischemic heart disease but limited atherosclerotic involvement [11,19,20].

An insertion/deletion polymorphism in intron 16 of the ACE gene have been shown to have major impact on the plasma angiotensin I-converting enzyme activity and accounted for 47% of the total phenotypic variance of serum ACE [21]. We have previously shown that D/D polymorphism of the ACE gene was significantly different between subjects with coronary artery disease and controls (p = 0.002) in Turkish population [12]. Recently, Völzke *et al*. demonstrated the effects angiotensin II type 1 receptor 1166A > C and angiotensinogen M235T gene polymorphisms to 2-year outcomes after CABG surgery [3]. However, Popov *et al*. showed that ACE-ins/del had no influence on mortality rate or perioperative systemic hemodynamic after CABG surgery [22]. Our findings with respect to ACE-ins/del polymorphisms demonstrated that D/D genotype had increased association with hypertension, peripheral vascular disease, and previous myocardial infarction but clinical outcomes did not differ between I/I, I/D, and D/D genotypes potentially due to the limited statistical power with respect to the end points of the present study.

The major limitations of this study are the sample size and the number of SNPs investigated which limits significant conclusions. Furthermore, our study population consists of Turkish patients, so these data cannot be generalized to other ethnic groups and populations. Polymorphism-association studies of cardiovascular disease such as this report should be interpreted with caution until they have been confirmed in other patient populations.

## List of abbreviations used

ACE-ins/del: angiotensin I-converting enzyme insertion/deletion; CABG: coronary artery bypass grafting surgery; CAD: coronary artery disease; CK-MB: creatine kinase isoenzyme; CPB: Cardiopulmonary bypass; D/D: deletion/deletion; EDTA: Ethylene-Diamine-Tetra-Acetic acid; FVL: factor V gene G1691A; ICU: intensive care unit; I/D: insertion/deletion; I/I: insertion/insertion; LVEF: left ventricular ejection fraction; PT G20210A: prothrombin/factor II G20210A; RT-PCR: real-time polymerase chain reaction; SNP: single nucleotide polymorphisms.

## Competing interests

This study was supported by a grant from Ankara University School of Medicine Research Council, Turkey. The authors state that they have no conflict of interests.

## Authors' contributions

OE, SD participated in the design of the study and drafted the manuscript. YE, YDA carried out the molecular genetic studies and the immunoassays, participated in the sequence alignment. CZ participated in the sequence alignment. ARA, UO, NA conceived of the study, participated in its design and coordination, helped to draft the manuscript and give final approval of the version to be published. All authors read and approved the final manuscript.
